# Caudal Regulates the Spatiotemporal Dynamics of Pair-Rule Waves in *Tribolium*

**DOI:** 10.1371/journal.pgen.1004677

**Published:** 2014-10-16

**Authors:** Ezzat El-Sherif, Xin Zhu, Jinping Fu, Susan J. Brown

**Affiliations:** 1 Genetics Program, Kansas State University, Manhattan, Kansas, United States of America; 2 Division of Biology, Kansas State University, Manhattan, Kansas, United States of America; New York University, United States of America

## Abstract

In the short-germ beetle *Tribolium castaneum*, waves of pair-rule gene expression propagate from the posterior end of the embryo towards the anterior and eventually freeze into stable stripes, partitioning the anterior-posterior axis into segments. Similar waves in vertebrates are assumed to arise due to the modulation of a molecular clock by a posterior-to-anterior frequency gradient. However, neither a molecular candidate nor a functional role has been identified to date for such a frequency gradient, either in vertebrates or elsewhere. Here we provide evidence that the posterior gradient of *Tc-caudal* expression regulates the oscillation frequency of pair-rule gene expression in *Tribolium*. We show this by analyzing the spatiotemporal dynamics of *Tc-even-skipped* expression in strong and mild knockdown of *Tc-caudal*, and by correlating the extension, level and slope of the *Tc-caudal* expression gradient to the spatiotemporal dynamics of *Tc-even-skipped* expression in wild type as well as in different RNAi knockdowns of *Tc-caudal* regulators. Further, we show that besides its absolute importance for stripe generation in the static phase of the *Tribolium* blastoderm, a frequency gradient might serve as a buffer against noise during axis elongation phase in *Tribolium* as well as vertebrates. Our results highlight the role of frequency gradients in pattern formation.

## Author Summary

One of the most popular problems in development is how the anterior-posterior axis of vertebrates, arthropods and annelids is partitioned into segments. In vertebrates, and recently shown in the beetle *Tribolium castaneum*, segments are demarcated by means of gene expression waves that propagate from posterior to anterior as the embryo elongates. These waves are assumed to arise due to the regulation of a molecular clock by a frequency gradient. However, to date, neither a candidate nor a functional role has been identified for such a frequency gradient. Here we provide evidence that a static expression gradient of *caudal* regulates pair-rule oscillations during blastoderm stage in *Tribolium*. In such a static setup, a frequency gradient is essential to convert clock oscillations into a striped pattern. We further show that a frequency gradient might be essential even in the presence of axis elongation as a buffer against noise. Our work also provides the best evidence to date that Caudal acts as a type of morphogen gradient in the blastoderm of short-germ arthropods; however, Caudal seems to convey positional information through frequency regulation of pair-rule oscillations, rather than through threshold concentration levels in the gradient.

## Introduction

The anterior-posterior (AP) axis of arthropods, annelids, and vertebrates is partitioned into segments. The French flag model, in which threshold concentrations of morphogen gradients are interpreted by downstream genes to partition a developing tissue [1], [2], provides the main theoretical framework explaining segmentation in *Drosophila*. Specifically, gradients of maternal factors span the AP axis of *Drosophila* providing positional information to downstream gap genes, which in turn diffuse in the syncytial blastoderm to form more localized morphogen gradients. Both maternal and gap gene gradients provide further positional information to the pair-rule genes whose striped expression is the first indication of segmentation in the embryo [3].

In *Drosophila*, all segments form more or less simultaneously in a syncytial blastoderm of fixed AP axis length. In contrast, vertebrate segmentation (somitogenesis) takes place sequentially in an elongating and cellularized embryo. A different model, the ‘clock and wavefront’ explains segmentation in vertebrates [4], [5]. Multiple genes (*hairy*/*enhancer-of-split* and genes of Notch, Wnt and FGF signaling pathways) show oscillatory expression in the presomitic mesoderm (PSM) of the vertebrate embryo and are thought to be constituents of a molecular clock [6], [7]. In cells located anterior to a wavefront, oscillations are arrested into stable stripes. The wavefront is thought to be defined by a moving threshold that forms within the overlapping posterior gradients of Wnt and FGF [8], [9] and an opposing retinoic acid gradient [10]. Oscillations seem to arrest gradually (i.e. they are modulated by a frequency gradient) as evidenced by kinematic expression waves that sweep the PSM from posterior to anterior [7].

In most short-germ arthropods, anterior segments form in a blastoderm, as in *Drosophila*, while posterior segments form subsequently during the germband stage out of a population of cells at the posterior end of the embryo (termed the ‘growth zone’) [11], reminiscent of somitogenesis in vertebrates. Although it is conceivable that short-germ arthropods utilize a ‘French flag’-based segmentation mechanism in the blastoderm and a ‘clock and wavefront’ mechanism in the germband, it has recently been shown that a segmentation clock operates in both the germband [12] and blastoderm [13] of the short-germ insect *Tribolium castaneum*, where waves of pair-rule gene expression (specifically *Tc-even-skipped* (*Tc-eve*)) propagate from posterior to anterior [13].

The identification of factors that provide positional information for segmentation in the blastoderm of short-germ arthropods has been controversial [14]–[18]. Demonstration of the clock-based nature of short-germ segmentation fuels this debate as attention now turns to the search for factors functioning as a wavefront. The homeodomain transcription factor Caudal (Cad) has been implicated as playing a prominent role in AP patterning in arthropods since its expression overlaps with the newly forming stripes [19]. Cad is required for segmentation in the *Drosophila* abdomen [20], and for posterior patterning in other species [21], [22]. It plays an even more prominent role in non-diptran insect segmentation; it is required for trunk segmentation in *Nasonia vitripennis* [23] and for both trunk and gnathal segmentation in *Tribolium castaneum* [24] and *Gryllus* bimaculatus [25]. However, the exact role of Cad in segmentation is still not known. Here we test the hypothesis that the posterior gradient of *Tribolium cad* (*Tc-cad*) expression regulates the oscillation frequency of pair-rule gene expression to produce kinematic waves in the *Tribolium* blastoderm. We found that the expression of *Tc-eve* was abolished in strong *Tc-cad* RNAi knock-down embryos, but in weak *Tc-cad* knock-down embryos, the *Tc-eve* expression domain was posteriorly shifted and its oscillation frequency reduced. Perturbing the *Tc-cad* gradient in different ways by knocking-down its regulators further demonstrated that the extension, intensity, and slope of the *Tc-cad* gradient correlated with the extension, frequency, and width of *Tc-eve* expression waves, respectively. As shown by computer simulations, these observations are consistent with the hypothesis that *Tc-cad* functions as a frequency gradient regulating the spatiotemporal dynamics of pair-rule gene oscillation in *Tribolium*. These observations, combined with the continued expression of *Tc-cad* in a posterior gradient suggest that *Tc-cad* also acts as a wavefront in the elongating germband. Our study highlights the concept of a frequency gradient as a pattern formation mechanism. Using computer modeling, we also showed that a graded frequency profile might even be essential within the clock-and-wavefront model as a buffer against noise.

## Results

### Characterizing *Tc-cad* expression in *Tribolium*

**Figure 1:**
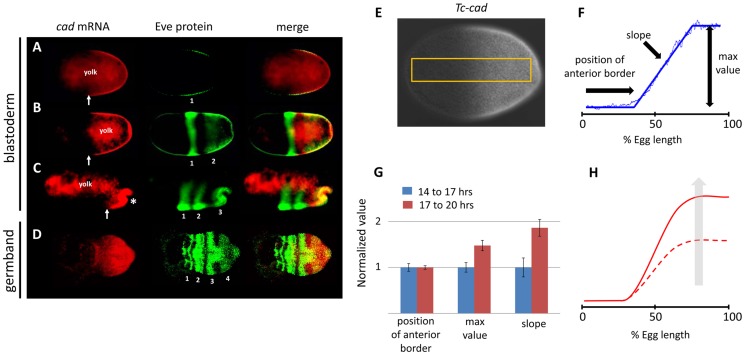
*Tc-cad* expression in *Tribolium*. (A–D) Concurrent *Tc-cad in situ* hybridization (red; first column) and Tc-EVE antibody staining (green; second column) were merged (third column) to show that *Tc-cad* expression overlaps with the emerging first two stripes of Tc-Eve in the blastoderm (A, B), and retreats to the posterior while the third stripe emerges (C). *Tc-cad* expression is confined in the growth zone during the germband stage to overlap with emerging stripes (fourth stripe in D). (E, F) Measuring *Tc-cad* expression across AP axis of the blastoderm (E, Text S3) and fitting raw measurements (thin blue line in F) to a linear-with-plateau curve (thick blue line in F) and calculating its three descriptors (F, Text S3). (G, H) As revealed by the change in the three descriptors of *Tc-cad* gradient over time (G), *Tc-cad* expression gradient builds up during 14–17 hours AEL but does not shift. *Tc-cad* dynamics are summarized in H; dashed curve: early, solid curve: late expression. Anterior to left. Error bars represent 95% confidence intervals.

The wave dynamics of *Tc-eve* in *Tribolium* can be explained by assuming a posterior-to-anterior gradient that positively regulates the frequency of *Tc-eve* oscillations [13]. *Tc-cad* is an obvious candidate to encode such a frequency gradient because its mRNA expression forms a posterior-to-anterior gradient that overlaps the *Tc-eve* expression waves arising at the posterior throughout *Tribolium* segmentation (Figure 1 A–D). Since studying segmentation in the germband phase of *Tribolium* development is hindered by the truncation phenotype generated by most segmentation gene knock-downs, we largely restricted our analysis to the stripes that form during the blastoderm stage. The expression of *Tc-cad* in the blastoderm (Figure 1 E) is approximated with reasonable accuracy by a posterior-to-anterior linear gradient that plateaus at the posterior end (Figure 1 F; Text S3). We used three descriptors to characterize this gradient: maximum posterior (plateau) value, position of anterior border, and slope (Figure 1 F). We analyzed the temporal dynamics of the *Tc-cad* gradient by calculating its three descriptors at 14–17 and 17–20 hours after egg lay (AEL) (Figure 1 G), spanning the formation of the first and second *Tc-eve* expression stripes in wild type (WT) [13] (analysis of later times was precluded by primitive pit formation, asterisk in Figure 1 C). As shown in Figure 1 G, the anterior border of *Tc-cad* expression gradient did not experience a significant shift during the formation of the first and second *Tc-eve* stripes, (which is also evident in Figure 1 A, B). However, both the maximum posterior value and the slope of the *Tc-cad* gradient increased over time. This indicates that the *Tc-cad* gradient was building up during the formation of the first and second *Tc-eve* stripes, but did not undergo a substantial shift along the AP axis (Figure 1 H). Characterizing *Tc-cad* gradient dynamics with higher temporal resolution (Figure S1) indicates that this buildup phase occurred between 14 to 16 hours AEL (i.e. before completion of the first *Tc-eve* stripe), after which the gradient was more or less static. This argues against a substantial influence of *Tc-cad* temporal dynamics on the wave dynamics of *Tc-eve* expression in the blastoderm. By the time the third stripe formed in the germ rudiment, the *Tc-cad* gradient had retreated toward posterior (Figure 1 C).

The spatial distribution of *Tc-cad* renders it a probable wavefront candidate in a clock-and-wavefront model. In the traditional model, a wavefront should retract posteriorly (like *Tc-cad* expression during the germband stage). However, a *static* but *smooth* gradient (like *Tc-cad* expression during the formation of first and second *Tc-eve* stripes in the blastoderm) that modulates the frequency of *Tc-eve* oscillation is, in principle, capable of forming a striped expression pattern (Movies S1, lower panel) [13], [26]. Taking the initial buildup phase of the *Tc-cad* gradient into consideration (Movies S1, upper panel) yields similar results. However, this buildup phase is expected to slow down the formation of the first stripe (Figure S2). This agrees with experiment, since the first cycle of *Tc-eve* oscillations starts at 13.5 hours AEL and ends at 17 hours AEL (i.e. the first stripe takes 3.5 hours to form), while the second cycle starts at 17 hours AEL and ends at 20 hours (i.e. the second stripe takes 3 hours to form) [13]. However, this is not obvious in the timing results presented here (see below), since we chose to start our analysis at 14 hours AEL.

### Regulation of the *Tc-cad* gradient

In both vertebrates and arthropods, canonical Wnt is a positive regulator of *cdx*/*cad* [25], [27]–[29]. Once bound by Wnt ligand, the receptor Frizzled recruits the β-catenin destruction complex (comprising Axin, APC, and other factors), rendering β-catenin free to enter the nucleus and bind Pangolin (TCF) with the help of Legless (Lgs), Pygopous (Pygo) and other coactivators [30] to activate Wnt targets. In *Tribolium*, *wnt1* and *wnt8* are expressed at the posterior pole of the blastoderm, and at the posterior end of the growth-zone in the germband [31], which is expected to produce a posterior gradient of Wnt activity, the formation of which is enhanced by the anterior localization of Wnt repressors in the blastoderm [28], [32].

**Figure 2:**
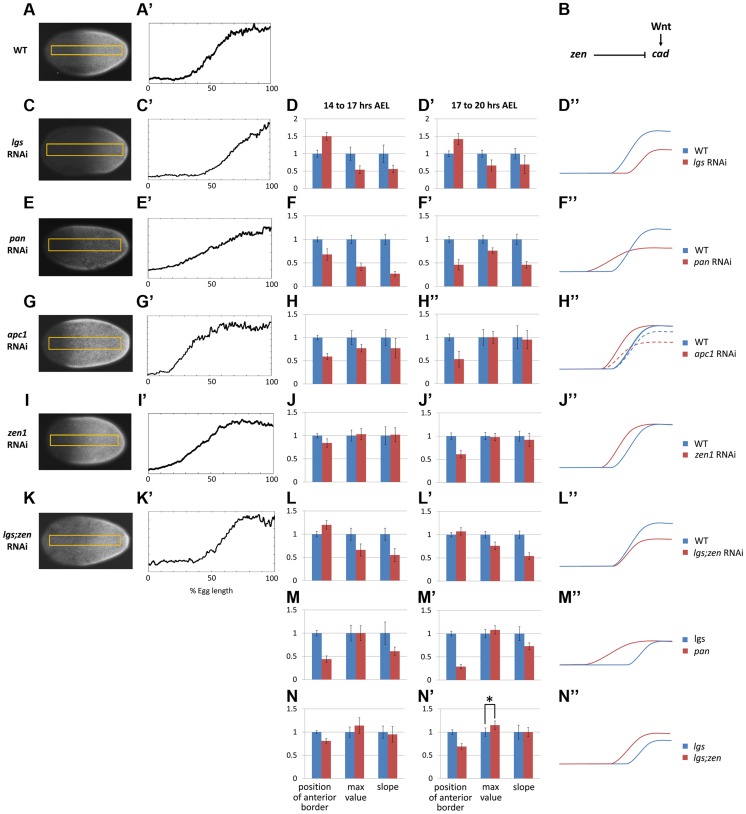
Characterization of *Tc-cad* gradient in WT and RNAi knockdowns. (A, A′) *Tc-cad* gradient in WT. (B) A model for *Tc-cad* regulation in the *Tribolium* blastoderm. (C–D″) *Tc-cad* gradient expression in a *Tc-lgs* RNAi embryo (C, C′), and the average of its three descriptors normalized to WT values (Text S3) in 14–17 AEL (D) and 17–20 AEL (D′). As inferred from (D, D′), a comparison between the spatial distribution of *Tc*-*cad* gradient in *Tc-lgs* RNAi embryos and that of WT is summarized in D″ (not to scale). The same was performed for *Tc-pan* (E–F″), *Tc-apc1* (G–H″; in H″: dashed curve for 14–17 AEL and solid curve for 17–20 AEL), *Tc-zen1* (I–J″), and *Tc-lgs*;*Tc-zen1* (K–L″) RNAi embryos. (M–M″) the average of the three descriptors of the *Tc-cad* expression gradient in *Tc-pan* RNAi normalized to *Tc-lgs* RNAi values (Text S3). (N–N″) the average of the three descriptors of the *Tc-cad* expression gradient in *Tc-lgs*;*Tc-zen1* RNAi normalized to *Tc-lgs* RNAi values. Anterior to the left. Error bars represent 95% confidence intervals. Asterisk (*) represents p-value<0.05.

Manipulating Wnt activity affected *Tc-cad* expression in the *Tribolium* blastoderm. Knocking down *Tc-lgs* (a positive Wnt regulator) by means of maternal RNAi (Methods) shifted the *Tc-cad* expression gradient posteriorly (compare Figure 2 C–C′ to Figure 2 A–A′). In addition, the posterior maximum value of *Tc-cad* and slope of the gradient were reduced in *Tc-lgs* RNAi embryos compared to WT (Figure 2 D–D″).

Knocking down *Tc-apc1* (a negative Wnt regulator) repositioned the *Tc-cad* gradient anteriorly (Figure 2 G–H″). Interestingly, the maximum posterior value of the *Tc-cad* expression gradient at 14–17 hours AEL was lower in *Tc-apc1* RNAi embryos than in WT embryos (Figure 2 H), but eventually reached WT levels by 17–20 hours AEL (Figure 2 H′). Thus, it appears that the *Tc-cad* expression gradient takes longer to mature in *Tc-apc1* RNAi than in WT embryos, which might be indicative of early negative Wnt regulation of *Tc-cad*.

Knocking down another Wnt regulator, *Tc-pan*, also perturbed the *Tc-cad* expression gradient. Pan, a component of the activator complex, also acts as a repressor in the absence of nuclear β-catenin [33]. Hence, we expected Wnt activity to be reduced posteriorly but increased anteriorly in *Tc-pan* RNAi embryos compared to WT, resulting in a shallower Wnt gradient across the blastoderm, and consequently a shallower *Tc-cad* gradient. As expected, the border of the *Tc-cad* gradient in *Tc-pan* RNAi embryos shifted anteriorly, the gradient reached a lower maximum posterior value, and the slope was lower compared to WT (Figure 2 E–F″).

In *Drosophila*, two Hox3 type genes are involved in early patterning: *bicoid* (*bcd*), which is expressed anteriorly and plays a major role in AP patterning, and *zerknüllt* (*zen*), which is expressed dorsally and specifies the amnioserosa [34]. *Tribolium* lacks *bcd* [17] but one of its *zen* homologs, *Tc-zen1*, is expressed both anteriorly and dorsally [35]. Anterior expression precedes dorsal expression and is suspected to play a role in AP patterning [36]. As shown in Figure 2 I–J″, the *Tc-cad* gradient in *Tc-zen1* RNAi embryos shifted anteriorly, but had the same slope and maximum posterior expression level as WT, indicating that *Tc-zen1* represses *Tc-cad* anteriorly (see Figure 2 B for a summary of *Tc-cad* regulation).

### *Tc-cad* gradient regulates *Tc-eve* waves in *Tribolium*

**Figure 3:**
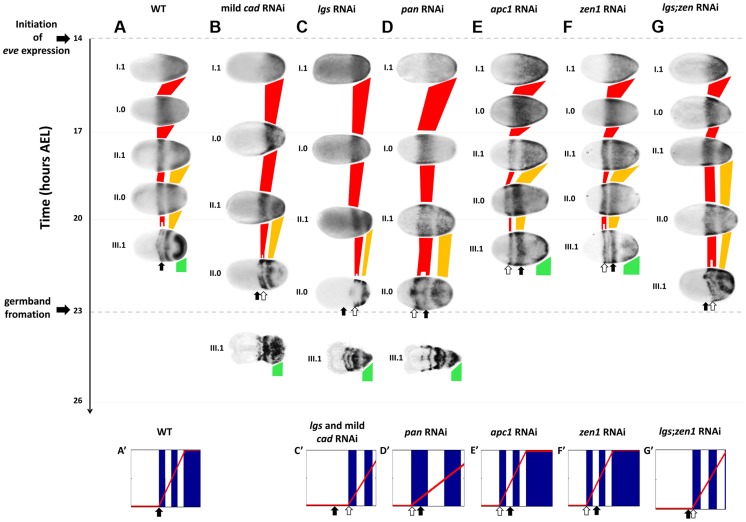
*Tc-eve* expression in WT and RNAi knockdowns. *Tc-eve* expression waves in WT (A), mild *Tc-cad* (B), *Tc-lgs* (C), *Tc-pan* (D), *Tc-apc1* (E), *Tc-zen1* (F) and *Tc-lgs*;*Tc-zen1* (G) RNAi embryos (First cycle/wave/stripe in red, second in gold, and third in green). *Tc-eve* expression patterns were classified according to the cycle of *Tc-eve* oscillation in the posterior end of the embryo (roman numerals) and the phase of the cycle (1 for high phase, and 0 for low; e.g. I.1: high phase of the first cycle). Embryos were mapped on the time axis according to timing data (see text). Arrows indicate the position of the anterior border of *Tc-eve* expression at 20–23 hours AEL in WT (black arrow) and in different knockdowns (white arrows). Shown also are snapshots of computer simulations of a *Tc-eve* oscillator the frequency of which is modulated by the *Tc-cad* gradient of WT (A′; see Movie S1, upper panel), mild *Tc-cad* and *Tc-lgs* RNAi (C′; see Movie S2, lower panel), *Tc-pan* RNAi (D′; see Movie S3, lower panel), *Tc-apc1* (E′; see Movie S4, lower panel), *Tc-zen1* (F′; see Movie S5, lower panel), and *Tc-lgs*;*Tc-zen1* (G′; see Movie S6, lower panel) RNAi embryos; blue: *Tc-eve* expression, red curve: *Tc-cad* expression gradient. Snapshots were taken at the end of the corresponding simulations. Anterior to the left. Simulations were performed using Matlab (code is available in Text S1).

In *Tribolium*, *Tc-eve* is expressed in waves that shrink while propagating from posterior to anterior (Figure 3 A) [13]. *Tc-eve* and *Tc-cad* RNAi embryo display similar phenotypes lacking all post oral segments, and previous studies implicate *cad* in the regulation of *eve* in arthropods [24], [25].

#### *Tc-cad* RNAi

**Figure 4:**
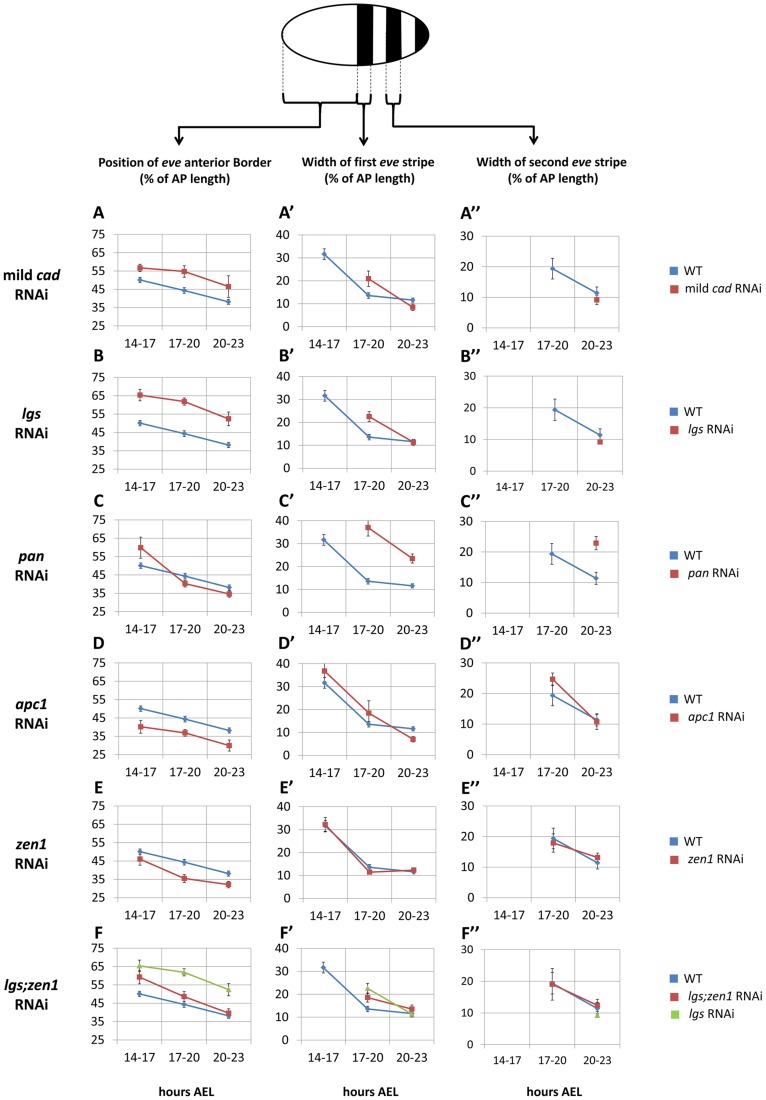
Spatial characteristics of *Tc-eve* waves over time in WT and RNAi knockdowns. (A, B, C, D, E, F) average position of the anterior border of *Tc-eve* expression over time in mild *Tc-cad* (A), *Tc-lgs* (B), *Tc-pan* (C), *Tc-apc1* (D), *Tc-zen1* (E) and *Tc-lgs*;*Tc-zen1* (F) RNAi embryos (red) compared to WT (blue; along with *Tc-lgs* RNAi in case of *Tc-lgs*;*Tc-zen1*, green). Same comparisons were performed for average width of first (A′, B′, C′, D′, E′, F′) and second (A″, B″, C″, D″, E″, F″) *Tc-eve* stripes. At top is a depiction of *Tc-eve* expression (black stripes) in a WT *Tribolium* embryo at late blastoderm stage; anterior to the left. All measurements were normalized to AP axis lengths (Text S3 and Figure S5). A missing data point for a certain stripe indicates that stripe has not formed yet; a stripe proper should have both anterior and posterior borders. Error bars represent 95% confidence intervals.

To examine a possible role of *Tc-cad* in regulating *Tc-eve*, we characterized the dynamics of *Tc-eve* expression in WT and *Tc-cad* RNAi embryos. Strong *Tc-cad* RNAi completely abolished *Tc-eve* expression (Figure S3 A). We produced milder effects by injecting lower concentrations of *Tc-cad* dsRNA. In these embryos, waves of *Tc-eve* expression propagated from posterior to anterior (Figure 3 B); however, the final positions of the *Tc-eve* stripes were shifted posteriorly compared to WT (compare Figure 3 B with Figure 3 A; Figure 4 A).

**Figure 5:**
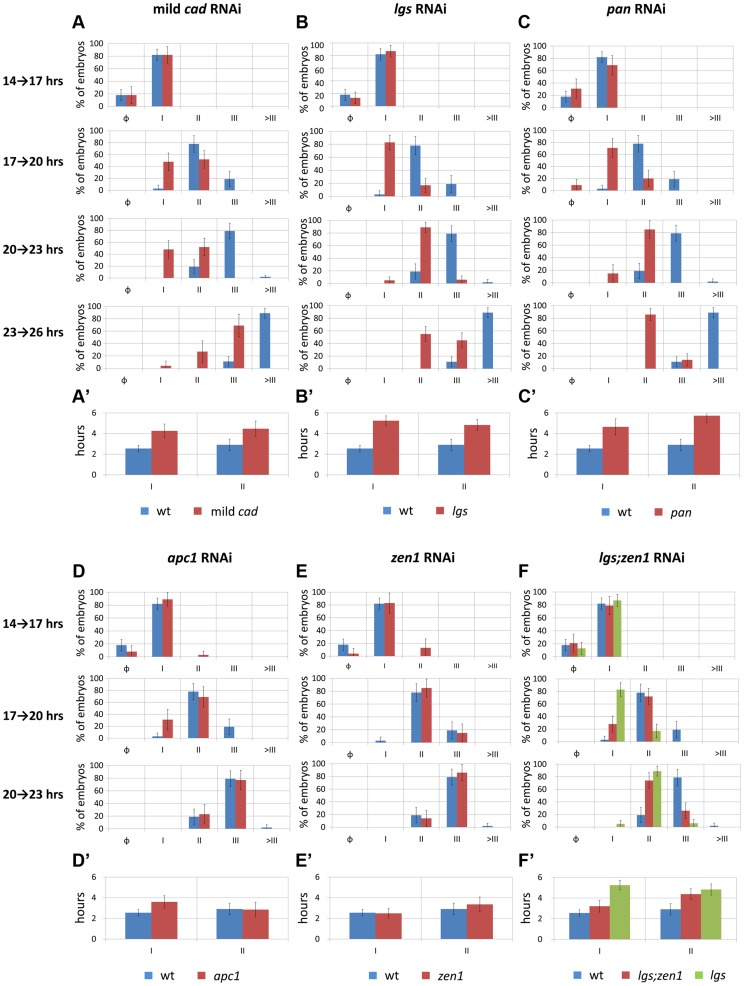
Temporal dynamics of *Tc-eve* expression at the posterior end of the embryo in WT and RNAi knockdowns. (A, B, C, D, E, F) percentage distributions of *Tc-eve* expression classes (classification was based on *Tc-eve* oscillation cycle in the posterior end, see Figure 3) in different timed egg collections in multiple RNAi knockdowns (red bars) in comparison with WT (blue bars): mild *Tc-cad* (A), *Tc-lgs* (B), *Tc-pan*(C), *Tc-apc1*(D), *Tc-zen1*(E), and *Tc-lgs*;*Tc-zen1*(F) RNAi embryos. Cycle I embryos are those going from high (phase I.1) to low (phase I.0) Tc-eve expression levels at the posterior end to from the first Tc-eve stripe (Figure 3); cycle II embryos are those going from high (phase II.1) to low (phase II.0) Tc-eve expression levels to form the second Tc-eve stripe (and so on). Class distributions were used to estimate the duration of different *Tc*-*eve* oscillation cycles (A′, B′, C′, D′, E′, and F′; see Text S3). Error bars represent 95% confidence intervals.

In the mild *Tc-cad* RNAi embryos, the three expected stripes did not fully form prior to germ rudiment condensation (Figure 3 B). To determine if this is due to a reduction in *Tc-eve* oscillation frequency, we measured the maximum frequency of *Tc-eve* oscillations by tracing *Tc-eve* expression over time at the posterior end of the blastoderm (Figure 5 A; Text S3). In WT, a new *Tc-eve* cycle peaked in every 3-hour egg collection (Figure 5 A, blue bars), consistent with the ∼3 hour periodicity we previously reported for *Tc-eve* oscillations at 23–24°C [13]. For mild *Tc-cad* RNAi, while cycle I initiated at 14 to 17 hrs AEL similar to WT, it persisted through 17 to 20 hrs AEL (Figure 5 A, red bar). The duration of *Tc-eve* cycles I and II in *Tc-cad* RNAi embryos (Figure 5 A′, Text S3) both lasted longer than in WT.

#### *Tc-lgs* RNAi

In *Tc-lgs* RNAi embryos, the anterior border of *Tc-cad* expression shifted posteriorly and the posterior maximum level decreased (Figure 2 C–D″). The *Tc-eve* waves were also shifted posteriorly, in accordance with the posterior shift of the *Tc-cad* gradient (compare Figure 3 C with Figure 3 A; Figure 4 B). In addition, the *Tc-eve* oscillation frequency was reduced (Figure 5 B, B′), corresponding to the reduction in posterior *Tc-cad* levels. Both the posterior shift and the reduced frequency of *Tc-eve* oscillations at the posterior end of the blastoderm upon the reduction of the *Tc-cad* gradient (either in mild *Tc-cad* RNAi or *Tc-lgs* RNAi) is predicted by a model in which the *Tc-cad* gradient regulates the frequency of *Tc-eve* oscillations (Movie S2, compare Figure 3 C′ to Figure 3 A′).

#### *Tc-pan* RNAi

In contrast, the *Tc-cad* gradient shifted anteriorly in *Tc-pan* RNAi embryos (Figure 2 E–F″). Correspondingly, *Tc-eve* waves were shifted anteriorly in *Tc-pan* RNAi compared to WT (compare Figure 3 D to Figure 3 A; Figure 4 C for 17–23 hours AEL). However, similar to *Tc-lgs* RNAi, *Tc-cad* mRNA levels were reduced at the posterior end of *Tc-pan* RNAi embryos (Figure 2 E–F″). The corresponding *Tc-eve* oscillation frequency was also reduced (Figure 5 C, C′). In addition to the anterior shift and frequency reduction of *Tc-eve* expression waves, the width of *Tc-eve* stripes in *Tc-pan* RNAi embryos was strikingly wider than those in WT (compare Figure 3 D to Figure 3 A; Figure 4 C′ and C″). This corresponds to the stretching effect of *Tc-pan* RNAi knock-down on the *Tc-cad* gradient, evident in the lower slope and anterior shift of this gradient in *Tc-pan* RNAi embryos compared to WT (Figure 2 F–F″).

Interestingly, in *Tc-lgs* RNAi embryos the first *Tc-eve* stripe, which formed at 17–20 hours AEL, was wider than that of WT (Figure 4 B′) in accordance with the reduction of the slope of *Tc-cad* gradient there (Figure 2 D–D″), but by 20–23 hours AEL the width of *Tc-eve* stripes is similar to WT (Figure 4 B′ and B″). In contrast to *Tc-pan* RNAi, *Tc-cad* slope reduction in *Tc-lgs* RNAi embryos might not be severe enough to result in detectable differences in the final width of *Tc-eve* stripes. Comparison of the *Tc-cad* gradient in RNAi embryos that were fixed and stained in parallel confirmed that while the level of *Tc-cad* expression at the posterior end in both *Tc-lgs* and *Tc-pan* was similar, the slope reduction in *Tc-pan* RNAi was more severe than in *Tc-lgs* RNAi embryos (Figure 2 M–M″).

The final anterior (but initial posterior) shift (Figure 4 C), the reduced frequency of *Tc-eve* oscillations at the posterior end of the blastoderm, and the wider *Tc-eve* stripe that were observed upon reducing and stretching the *Tc-cad* gradient in *Tc-pan* RNAi embryos is predicted by a model in which the *Tc-cad* gradient modulates the frequency of *Tc-eve* oscillations (Movie S3, Figure 3 D′).

#### *Tc-apc1* RNAi

In *Tc-apc1* RNAi embryos, *Tc-eve* waves shifted towards the anterior (compare Figure 3 E to Figure 3 A; Figure 4 D) corresponding to the anterior shift in the *Tc-cad* gradient (Figure 2 G–H″). The first *Tc-eve* stripe took longer to form in *Tc-apc1* RNAi embryos compared to WT (Figure 5 D′), corresponding to a lower maximum posterior value of *Tc-cad* in *Tc-apc1* RNAi embryos during 14–17 AEL (Figure 2 H). The second stripe formed with near normal kinetics in *Tc-apc1* RNAi embryos (Figure 5 D′), in accordance with the eventual increase of the maximum posterior value of *Tc-cad* in *Tc-apc1* RNAi during 17–20 AEL (Figure 2 H′).

#### *Tc-zen1* RNAi

In *Tc-zen1* RNAi embryos, *Tc-eve* waves shifted towards the anterior (compare Figure 3 F to Figure 3 A) corresponding to the anterior shift of the *Tc-cad* gradient (Figure 2 I–J″). The buildup of *Tc-cad* transcripts in the posterior in *Tc-zen1* RNAi embryos was similar to those in WT (Figure 2 J–J″); correspondingly, the timing of *Tc-eve* waves in *Tc-zen1* RNAi and WT embryos are very similar (Figure 5 E, E′). The anterior shift of *Tc-eve* waves upon anterior extension of the *Tc-cad* gradient (in *Tc-apc1* and *Tc-zen1* RNAi) is predicted by a model in which *Tc-cad* gradient modulates the frequency of *Tc-eve* oscillations (Movies S4 and S5; Figures 3 E′ and 3 F′).

The slope of *Tc-cad* gradient in both *Tc-apc1* and *Tc-zen1* RNAi embryos is largely similar to that of WT (Figure 2 H–H″ and J–J″), and the corresponding width of *Tc-eve* stripes is also similar to WT (Figure 4 D′, D″, E′ and E″), with the possibility of a slight initial reduction in the slope of the *Tc-cad* gradient in *Tc-apc1* RNAi embryos (Figure 2 H) and the corresponding slight increase in *Tc-eve* stripe width (Figure 4 D′). The final stripe width reduction (at 20–23 hours AEL) in *Tc-apc1* RNAi embryos (and possibly *Tc-cad* RNAi embryos; Figure 4 A′ and D′) could be due to a defect in the characteristic split of mature *Tc-eve* stripes into secondary, segmental stripes (compare Figure 3 E class III.1 embryo to Figure 3 A class III.1 embryo; while the splitting defect is variable in mild *Tc-cad* RNAi embryos, Figure S3 B).

#### *Tc-lgs*;*Tc-zen1* double RNAi

Since *Tc-lgs* and *Tc-zen1* RNAi shifted the *Tc-cad* gradient (and *Tc-eve* stripes) in opposite directions, we sought to examine the effect of the double *Tc-lgs*;*Tc-zen1* RNAi knock-down. *Tc-zen1* RNAi rescued to some degree the posterior shift in *Tc-cad* gradient induced by *Tc-lgs* RNAi (Figure 2 K–L″). The anterior border of the *Tc-eve* expression domain in *Tc-lgs*;*Tc-zen1* double RNAi embryos is closer to that of WT than that of *Tc-lgs* RNAi (Figure 3G; Figure 4 F). Surprisingly, although the *Tc-cad* posterior expression level is not altered in *Tc-zen1* RNAi, the posterior maximum expression level of *Tc-cad* was partially rescued in *Tc-lgs*;*Tc-zen1* double RNAi embryos at 17–20 hours AEL (Figure 2 N–N″). Corresponding to this, The first *Tc-eve* stripe forms more quickly in *Tc-lgs*;*Tc-zen1* RNAi compared to *Tc-lgs* RNAi (Figures 5 F, F′). However, this rescue effect eventually fades by the end of the blastoderm stage (20 to 23 hours AEL; Figure 5 F, F′), when *Tc-zen1* is normally down-regulated (Figure S4).

The intermediate phenotype of *Tc-lgs*;*Tc-zen1* RNAi between that of WT and *Tc-lgs* RNAi is predicted by a model in which *Tc-cad* gradient modulates the frequency of *Tc-eve* oscillations (Movie S6; Figure 3 G′).

### Graded frequency profile as a buffer against noise

Axis elongation is an essential component of the clock-and-wavefront model. We have previously shown that blastoderm segmentation in *Tribolium* seems to be clock-based [13]. Despite the lack of axis elongation at the blastoderm stage, we did not exclude the possible existence of a retreating frequency gradient (wavefront). In the current study, we provide evidence that *Tc-cad* expression acts as a frequency gradient that modulates pair-rule gene oscillations in the blastoderm. Although a static step frequency gradient (i.e. suddenly dropping from non-zero to zero frequency) does not possess any patterning capacity, a static but gradually decreasing frequency gradient can generate a striped pattern [26]. Indeed, the first two stripes of *Tc-eve* form during a time period when the *Tc-cad* gradient is largely static. After the formation of the first two stripes, *Tc-cad* expression then abruptly retreats to the prospective growth zone (Figure 1 C). Later during axis elongation in the germband stage, *Tc-cad* expression retreats posteriorly with every newly forming *Tc-eve* stripe (Figure 1 D).

**Figure 6:**
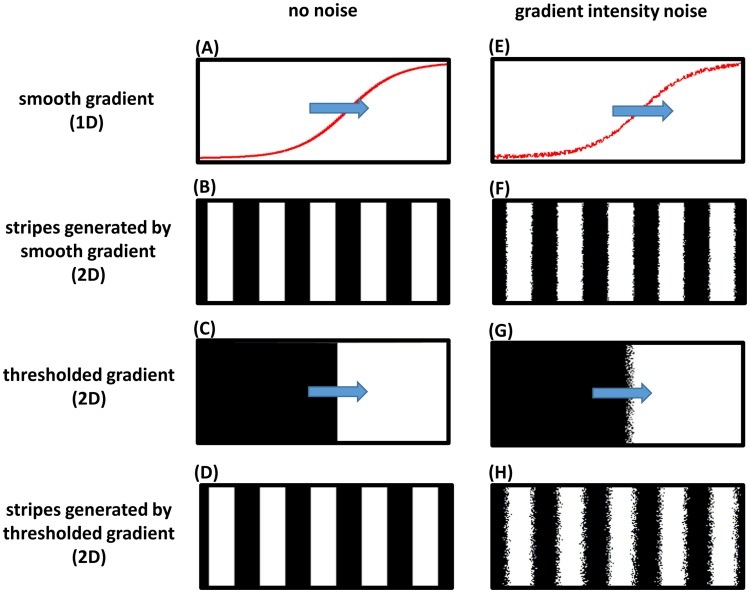
Frequency profile and robustness of the clock-and-wavefront model. A computer simulation of a two-dimensional (2D) lattice of oscillators (horizontal and vertical axes of the lattice represent the AP and lateral axes of the embryo, respectively; posterior to the right). Each oscillator runs independently with a frequency determined by a smooth spatial gradient that retracts from anterior to posterior, with or without applying a threhold. (A) one-dimensional (1D) lateral cross section of the 2D smooth gradient (see Movies S7 for the 2D version; direction of movement is shown in blue arrow). (B) stripes generated if the smooth gradient shown in (A) is directly applied to the oscillators lattice (high phase of oscillation is shown in white and low phase in black); see Movie S7. (C) a 2D thresholded version of the smooth gradient shown in (A). (D) stripes generated if the thresholded gradient shown in (C) is applied to oscillators lattice; see Movie S8. (E–H) are the same as (A–D) after adding noise to frequency gradient intensity; see Movies S9 and S10. Simulations were performed using Matlab (code is available in Text S2).

However, in principle, a step frequency gradient is capable of generating a striped pattern during the germband retraction phase. In vertebrates, a transition from high to low frequency (termed the ‘arrest front’) is thought to be determined by a threshold within a retracting posterior gradient. Such a mechanism might be very sensitive to the location of the threshold. Uncertainty in threshold location due to noise might lead to the generation of noisy patterns. On the other hand, gradually arresting oscillations would average out the noise and make the mechanism independent of precise threshold location. To investigate this, we developed two computer models for the clock-and-wavefront mechanism: one that utilizes a step frequency gradient by applying a threshold on a retracting smooth gradient (threshold-based model), and the other utilizes a smooth retracting frequency gradient without applying any thresholds (threshold-free model). Both generated similar striped patterns in the absence of noise (Figures 6 A–D; Movies S7 and S8). We then investigated the performance of both models after introducing random fluctuations in the intensity of the posterior gradient at each cell. This is expected to result in independent random shifts in threshold locations across the lateral axis of the embryo, which ultimately leads to salt-and-pepper noise at the stripe borders; however, the threshold-free model is more robust to this type of noise than the threshold-based model (Figures 6 E–H; Movies S9 and S10).

## Discussion

In this work we provide evidence that an anterior-to-posterior gradient of *Tc-cad* expression in *Tribolium* regulates waves of *Tc-eve* pair-rule gene expression. By examining the spatiotemporal dynamics of *Tc-eve* expression in WT and RNAi knockdowns of different *Tc-cad* regulators, three correlations were revealed: (1) the spatial extent of *Tc-cad* correlates with that of *Tc-eve* waves, (2) the level of *Tc-cad* expression correlates with the frequency of *Tc-eve* oscillations at the posterior end of the blastoderm, and (3) the slope of the *Tc-cad* gradient correlates with the width of *Tc-eve* stripes. These three correlations are consistent with the hypothesis that the *Tc-cad* gradient modulates the frequency of pair-rule oscillations resulting in waves of gene expression (Figure 3 A′, C′–G′; Movies S2, S3, S4, S5, S6). A clock regulated by a frequency gradient is one way of transforming a temporally periodic process into a spatially periodic one; another would be the clock-and-wavefront model. One advantage of patterning with a frequency gradient, in contrast to the clock-and-wavefront model, is that it does not require axis elongation, which might explain how the *Tribolium* blastoderm is segmented. Another advantage, that we demonstrated using computer modelling, is that even within the framework of the clock-and-wavefront, utilizing a graded frequency profile renders the segmentation process more robust against noisy wavefront gene expression (Figure 6; Movies S7, S8, S9, S10).

### The role of Caudal in segmentation

In *Drosophila*, maternal *cad* mRNA (*Dm-cad*) is ubiquitously expressed in the early blastoderm [37]. A posterior-to-anterior protein gradient of Dm-Cad forms due to translational repression by a reciprocal gradient of Dm-Bicoid [38]. Dm-Cad acts as an activator of posterior gap [39] and pair-rule genes [40] and binds to the enhancers of these genes [41], [42]. However, the mild segmentation defects in embryos in which the shape of Dm-Cad gradient has been altered argues against its function as a morphogen gradient [20], [43]. In the wasp *Nasonia vitripennis*, *Nv-cad* plays a more prominent role in activating gap and pair-rule genes, and a limited positioning role [23]. In the cricket *Gryllus bimaculatus*, *Gb-cad* was found to activate the pair-rule gene *Gb-eve*, and activate and position gap gene domains. This indicates that *cad* might act as a morphogen gradient in non-dipteran insects. In this study, we described similar results in *Tribolium*. We showed that in strong *Tc-cad* RNAi, expression of *Tc-eve* was abolished (Figure S2 A); while in weak *Tc-cad* RNAi, *Tc-eve* expression was posteriorly shifted (Figure 3 B). However, a morphogen gradient acting through concentration thresholds is less likely to act in positioning the highly dynamic pair-rule gene expression domains in *Tribolium*. Instead, we argue that *Tc-cad* regulates the frequency of a pair-rule clock to produce the observed wave dynamics.

Three *cad* homologs are found in mouse: *Cdx1*, *Cdx2*, and *Cdx4*. They are expressed in nested domains in the posterior end of the embryo. The Cdx1–Cdx2 double mutant exhibits fused somites [44], suggesting a role in somitogenesis. However, the Cdx1–Cdx2 double mutant also shows down-regulation of some caudalizing factors involved in somitogenesis (such as *wnt3a*) that are themselves *Cdx* regulators [45], [46]. Cdx genes also directly regulate Hox genes in a dose dependent manner [47], [48], and even regulate their activation times [49].

In summary, *cad*(-*related*) genes are involved in posterior patterning in many species. While it is not clear whether they play a permissive or instructive role, there is evidence that they might act as a morphogen gradient for gap genes in basal insects (like in *Gryllus*) and for Hox genes in vertebrates. In this study, we showed that *Tc-cad* regulates the spatiotemporal dynamics of *Tribolium* pair-rule genes in a dose dependent manner, stressing the instructive role of *cad* in the development of a non-dipteran insect. However, we cannot exclude the possibility that *Tc-cad* regulates pair-rule genes indirectly. Indeed, *Tc-cad* and Wnt might cross-regulate in a positive feedback loop to form identical gradients. In this case, it is hard to decide which is the direct regulator (or whether both Wnt and Tc-Cad are direct regulators) of *Tc-eve* expression without performing detailed *cis*-regulatory analysis of the *Tc-eve* locus.

### The patterning capacity of frequency gradients and the robustness of the clock-and-wavefront model

In the original formulation of the clock-and-wavefront model, the anterior-to-posterior movement of a step frequency profile (i.e. suddenly dropping from non-zero to zero frequency) over an oscillating field of cells sequentially generates a striped pattern in an anterior-to-posterior order [4]. Later, this mechanism was modified by assuming a graded frequency profile to accommodate the observation that oscillations organize into kinematic waves in the chick PSM [7]. Several efforts have been made to identify molecular gradient(s) that regulate the frequency of the vertebrate segmentation clock. A posterior-to-anterior Wnt activity gradient was found to define the PSM oscillation domain in the mouse [50], [51]. Furthermore, down-regulation of Wnt activity reduced the clock frequency in both mouse and chick [52]. However, elevated and flattened constitutive stabilization of β-catenin in the mouse PSM only extended the oscillation domain, arguing against a role for the shape of Wnt activity gradient in segmentation [50]. A posterior-to-anterior FGF gradient in the PSM was found to define where oscillations arrest [9], [53], [54], but manipulating the level of FGF signaling does not alter the clock period [9], [52]. A gradient of Her13.2 in zebrafish was suggested to modulate clock frequency through heterodimerization with other zebrafish clock constituents: Her1 and Her7 [55], [56]. However, this idea was recently challenged and an alternative model of gradual switching between two oscillatory modules was suggested [57].

It is not known whether the gradual arrest of oscillations and the resulting kinematic waves in vertebrates have any functional role or are a mere peculiarity, since, based on computer simulations of the clock-and-wavefront model, stripe widths depend only on the wavefront velocity and the maximum clock period, not on the shape of the frequency profile [5]. Although used for cosmetic means within the clock-and-wavefront model, a graded frequency profile (even a static one) by itself has a patterning capacity [26]; kinematic waves were observed in an oscillating Zhabotinskii chemical reaction, where a reactant controlling the frequency of oscillation is distributed in a gradient [58], [59]. Since a static step frequency profile is unable to generate any stripes, the patterning capacity of a graded frequency profile might explain how blastodermal *Tc-eve* stripes in *Tribolium* form in the absence of axis elongation. Although the possibility of a yet unidentified frequency gradient that sweeps across the blastoderm still exists, we showed in this study that a strong candidate for the frequency gradient in *Tribolium*, *Tc-cad*, does not appreciably shift during the formation of the first two *Tc-eve* stripes (Figure 1 G, H).

In addition to its necessity in the absence of axis elongation, a graded frequency profile renders the clock-and-wavefront robust against noise in wavefront gene expression, as shown by computer simulations (Figure 6 and Movies S7, S8, S9, S10). This improvement in robustness might be due to the distributed nature by which oscillations are arrested in a graded frequency profile, in contrast to the total reliance on a single threshold in a step frequency profile. This and other recent works reinforce the importance of the concept of a frequency (or phase) gradient in sequential patterning [60], [61].

In clock-based segmentation models that utilize a static frequency gradient, stripes continue to shrink and never stabilize (although stripe shrinkage rate decreases with time, Movie S1). Stripe stabilization can be achieved by the retraction of the frequency gradient, kick-starting another ‘stabilizing’ genetic program that completely freezes the stripes. Such a stabilizing program might further refine the stripes and/or split them into secondary stripes. Interestingly, in the germband stage (where *Tc-cad* retracts continuously along with germband elongation), once a *Tc-eve* stripe forms, it splits into two secondary (segmental) stripes [13], whereas in the blastoderm stage, the first *Tc-eve* stripe does not split until *Tc-cad* expression completely retreats towards the posterior, at which time the second *Tc-eve* stripe is already formed and the third stripe is starting to emerge (Figure 1 B–D). This suggests a link between *Tc-cad* retraction and *Tc-eve* splitting. Stabilizing and refinement/splitting strategies might rely on auto- and cross-regulatory interactions between pair-rule genes or on a reaction diffusion mechanism [62] or both.

## Materials and Methods

### *In situ* hybridization, immunocytochemistry, and RNAi

*In situ* hybridization was performed using DIG-labeled RNA probes and anti-DIG::AP antibody (Roche). Signal was developed using NBT/BCIP (BM Purple, Roche), or Fast Red/HNPP (Roche). Immunocytochemistry was performed using anti-Eve (mouse monoclonal antibody 2B8, hybridoma bank, University of Iowa) as primary, and anti-mouse::POD as secondary antibody (ABC kit, Vector). AlexaFluor 488 tyramide (Invitrogen) was used to give green fluorescent signal. All expression analyses were performed using embryos from uninjected GA-1 strain (WT) or adult GA-1 females injected with double-stranded RNA (ds RNA) of the gene of interest. dsRNA was synthesized using the T7 megascript kit (Ambion) and mixed with injection buffer (5 mM KCl, 0.1 mM KPO_4_, pH 6.8) before injection. Used dsRNA concentrations: 200 ng/µl for severe *Tc-cad*, 7.5 ng/µl for mild *Tc-cad*, 200 ng/µl for *Tc-lgs*, 200 ng/µl for *Tc-pan*, 1 µg/µl for *Tc-apc1*, 1 µg/µl for *Tc-zen1*, and 200 ng/µl; 1 µg/µl for *Tc-lgs*;*Tc-zen* double RNAi.

### Egg collections for developmental time windows

One hour developmental windows were generated by incubating one hour egg collections at 23–24°C for the desired length of time. For 3-hour developmental windows, eggs were collected after three hours instead of one hour. The beetles were reared in whole-wheat flour supplemented with 5% dried yeast.

## Supporting Information

Figure S1 Detailed temporal dynamics of *Tc-cad* expression gradient in the blastoderm. Shown is the three descriptors of *Tc-cad* expression gradient in one-hour timed egg collections (Text S3) spanning the time period 14 to 20 hours AEL. *Tc-cad* expression gradient builds up during 14–16 hours AEL (but without appreciable AP shift). During 16–19 hours AEL, the gradient is more or less static, but starts to drop after 19 hours AEL. Error bars represent 95% confidence intervals.
(PDF)

Figure S2 Stripes form slower during the buildup phase of the frequency gradient. Shown are the oscillation dynamics over time of a point at the posterior end (far right) in the computer simulations shown in (A) upper panel and (B) lower panel of Movie S1.
(PDF)

Figure S3 *Tc-eve* in severe and mild *Tc-cad* knockdowns. (A) Shown are two embryos with comparable stage (flattened posterior stage); *Tc-eve* is expressed in two stripes in WT while its expression is abolished in strong *Tc-cad* RNAi. (B) In mild *Tc-cad* RNAi, *Tc-eve* stripes split into two secondary stripes (upper embryo; similar to WT; see class III.1 embryo in Figure 3 A) in some embryos, while in other embryos they do not (lower embryo). Anterior to left.
(PDF)

Figure S4 **Early and late** ***Tc-zen1*** **expression in** ***Tribolium*** **blastoderm.** The dorsal anterior expression of *Tc-zen1* (A) is down-regulated at the end of blastoderm stage (B) in WT *Tribolium* embryos.
(PDF)

Figure S5 Average AP axis lengths over time for WT and RNAi knockdowns. For 14–17, 17–20, 20–23 hours AEL egg collections, the average AP axis lengths were calculated and normalized to 14–17 hours AEL average value for WT, mild *Tc-cad*, *Tc-lgs*, *Tc-pan*, *Tc-apc1*, *Tc-zen1*, and *Tc-lgs;Tc-zen1* RNAi.
(PDF)

Movie S1 Modeling *Tc-eve* waves in WT. *Tc-eve* expression (blue) in the blastoderm was modeled by an array of oscillators along the horizontal axis (representing the AP axis; posterior to the right). Each oscillator runs independently with a frequency determined by a spatial gradient (red). Simulations were run using a frequency gradient (red) corresponding to *Tc-cad* in WT. In lower panel, the frequency gradient is static. In upper panel, the frequency gradient builds up exponentially to steady state values equal to that in lower panel. Simulations were performed using Matlab (code is available in Text S1).
(WMV)

Movie S2 Modeling *Tc-eve* waves in *Tc-lgs* RNAi embryos versus WT. Simulation with a frequency gradient corresponding to *Tc-cad* in WT (upper panel, which is similar to the upper panel of Movie S1) was contrasted to a simulation run using a frequency gradient corresponding to *Tc-cad* in *Tc-lgs* RNAi (posteriorly shifted with reduced posterior value and small decrease in slope, compared to WT). Simulations were performed using Matlab (code is available in Text S1).
(WMV)

Movie S3 Modeling *Tc-eve* waves in *Tc-pan* RNAi embryos versus WT. Same as in Movie S2, but with a frequency gradient corresponding to *Tc-cad* in WT compared with simulations run using a frequency gradient corresponding to *Tc-cad* in *Tc-pan* RNAi (anteriorly shifted with reduced posterior value and large decrease in slope, compared to WT).
(WMV)

Movie S4 Modeling *Tc-eve* waves in *Tc-apc1* RNAi embryos versus WT. Same as in Movie S2, but with a frequency gradient corresponding to *Tc-cad* in WT compared with simulations run using a frequency gradient corresponding to *Tc-cad* in *Tc-apc1* RNAi (anteriorly shifted with the same posterior value and slope as WT, but with slower buildup dynamics).
(WMV)

Movie S5 Modeling *Tc-eve* waves in *Tc-zen1* RNAi embryos versus WT. Same as in Movie S2, but with a frequency gradient corresponding to *Tc-cad* in WT compared with simulations run using a frequency gradient corresponding to *Tc-cad* in *Tc-zen1* RNAi (anteriorly shifted with the same posterior value, slope and buildup dynamics as WT).
(WMV)

Movie S6 Modeling *Tc-eve* waves in *Tc-lgs*;*Tc-zen1* double RNAi embryos versus WT. Same as in Movie S2, but with a frequency gradient corresponding to *Tc-cad* in WT compared with simulations run using a frequency gradient corresponding to *Tc-cad* in *Tc-lgs*;*Tc-zen1* RNAi (the anterior border is located between those of WT and *Tc-lgs* RNAi; the posterior value is higher than that of *Tc-lgs* RNAi but lower than that of WT; slope as *Tc-lgs* RNAi and buildup dynamics as WT and *Tc-lgs* RNAi).
(WMV)

Movie S7 Performance of the threshold-free model in the absence of noise. Shown is a computer simulation of 2D lattice of oscillators (horizontal and vertical axes of the lattice represent the AP and lateral axes of the embryo, respectively; posterior to the right). Each oscillator (which high phase output is shown in white and low phase in black; lowermost panel) runs independently with a frequency determined by a smooth spatial gradient (shown in greyscale: the brighter the higher the gradient intensity, uppermost panel) that retracts posteriorly with time. Shown is a version of the model that utilizes the smooth gradient to regulate frequency directly (threshold-free model). Simulations were performed using Matlab (code is available in Text S2).
(WMV)

Movie S8 Performance of the threshold-based model in the absence of noise. Same as in Movie S6, but with a version of the model that applies a threshold to the frequency gradient (threshold-based model; the thresholded gradient is shown in the middle panel).
(WMV)

Movie S9 Performance of the threshold-free model subjected to gradient intensity noise. Same as in Movie S6, but with the threshold-free model subjected to gradient intensity noise.
(WMV)

Movie S10 Performance of the threshold-based model subjected to gradient intensity noise. Same as in Movie S7, but with the threshold-based model subjected to gradient intensity noise.
(WMV)

Text S1 Matlab code for Movies S1, S2, S3, S4, S5, S6.
(DOCX)

Text S2 Matlab code for Movies S7, S8, S9, S10.
(DOCX)

Text S3 Supplemental experimental procedures.
(DOCX)
