# Supplementary figures and images for: Caudal Regulates the Spatiotemporal Dynamics of Pair-Rule Waves in Tribolium
Source: PLoS Genet. 2014 Oct 16;10(10):e1004677. doi: 10.1371/journal.pgen.1004677 (PMC4199486; doi:10.1371/journal.pgen.1004677)

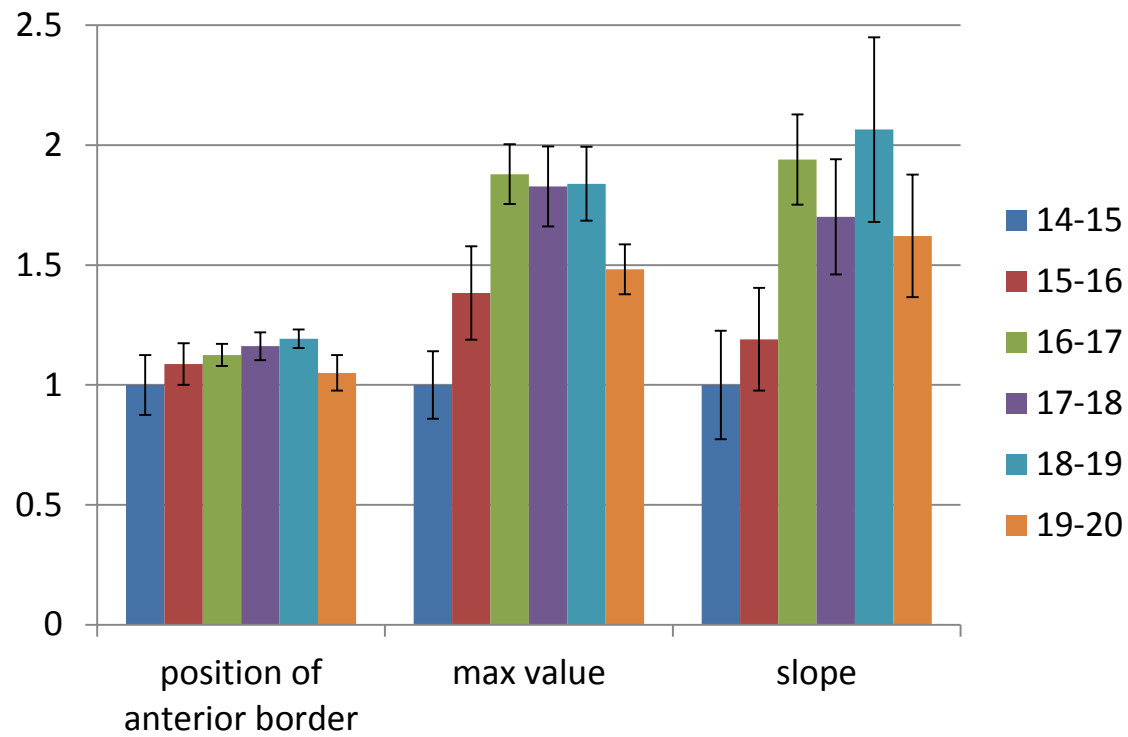

Supplement: Figure S1 — Detailed temporal dynamics of Tc-cad expression gradient in the blastoderm. Shown is the three descriptors of Tc-cad expression gradient in one-hour timed egg collections (Text S3) spanning the time period 14 to 20 hours AEL. Tc-cad expression gradient builds up during 14–16 hours AEL (but without appreciable AP shift). During 16–19 hours AEL, the gradient is more or less static, but starts to drop after 19 hours AEL. Error bars represent 95% confidence intervals. (PDF) [file pgen.1004677.s001.pdf]

(A)

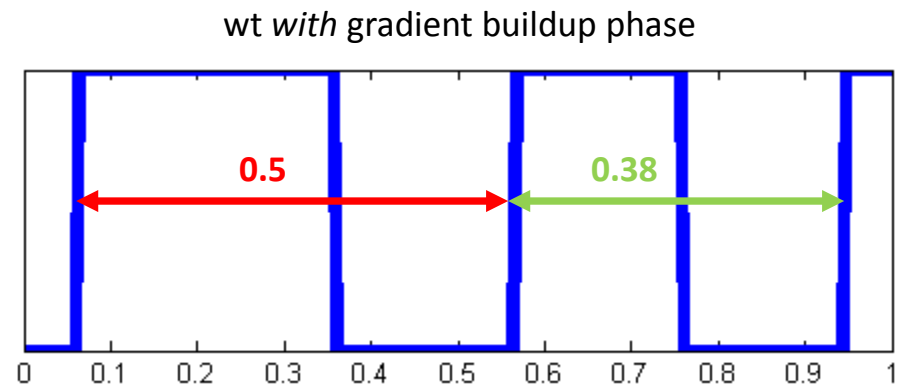

(B)

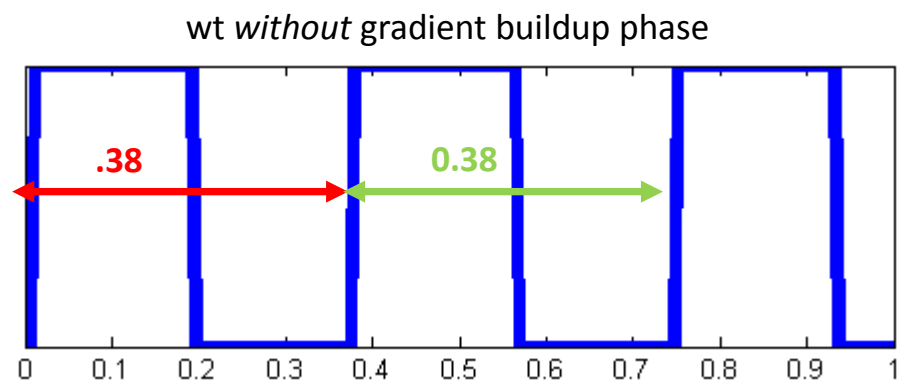

Time  
(unit time = total simulation time)

Supplement: Figure S2 — Stripes form slower during the buildup phase of the frequency gradient. Shown are the oscillation dynamics over time of a point at the posterior end (far right) in the computer simulations shown in (A) upper panel and (B) lower panel of Movie S1. (PDF) [file pgen.1004677.s002.pdf]

**A**

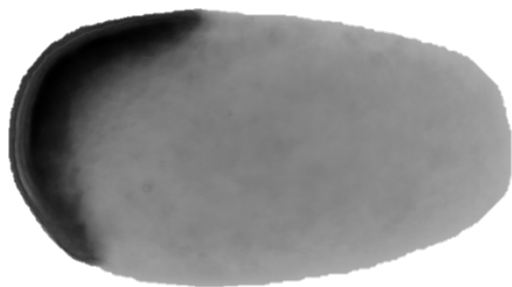

**B**

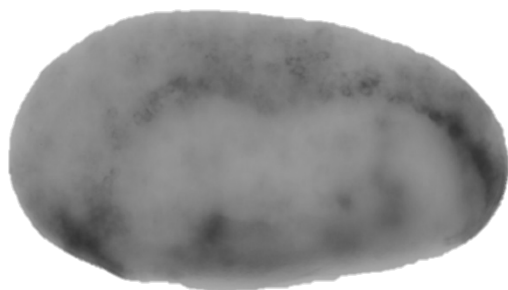

Supplement: Figure S4 — Early and late Tc-zen1 expression in Tribolium blastoderm. The dorsal anterior expression of Tc-zen1 (A) is down-regulated at the end of blastoderm stage (B) in WT Tribolium embryos. (PDF) [file pgen.1004677.s004.pdf]

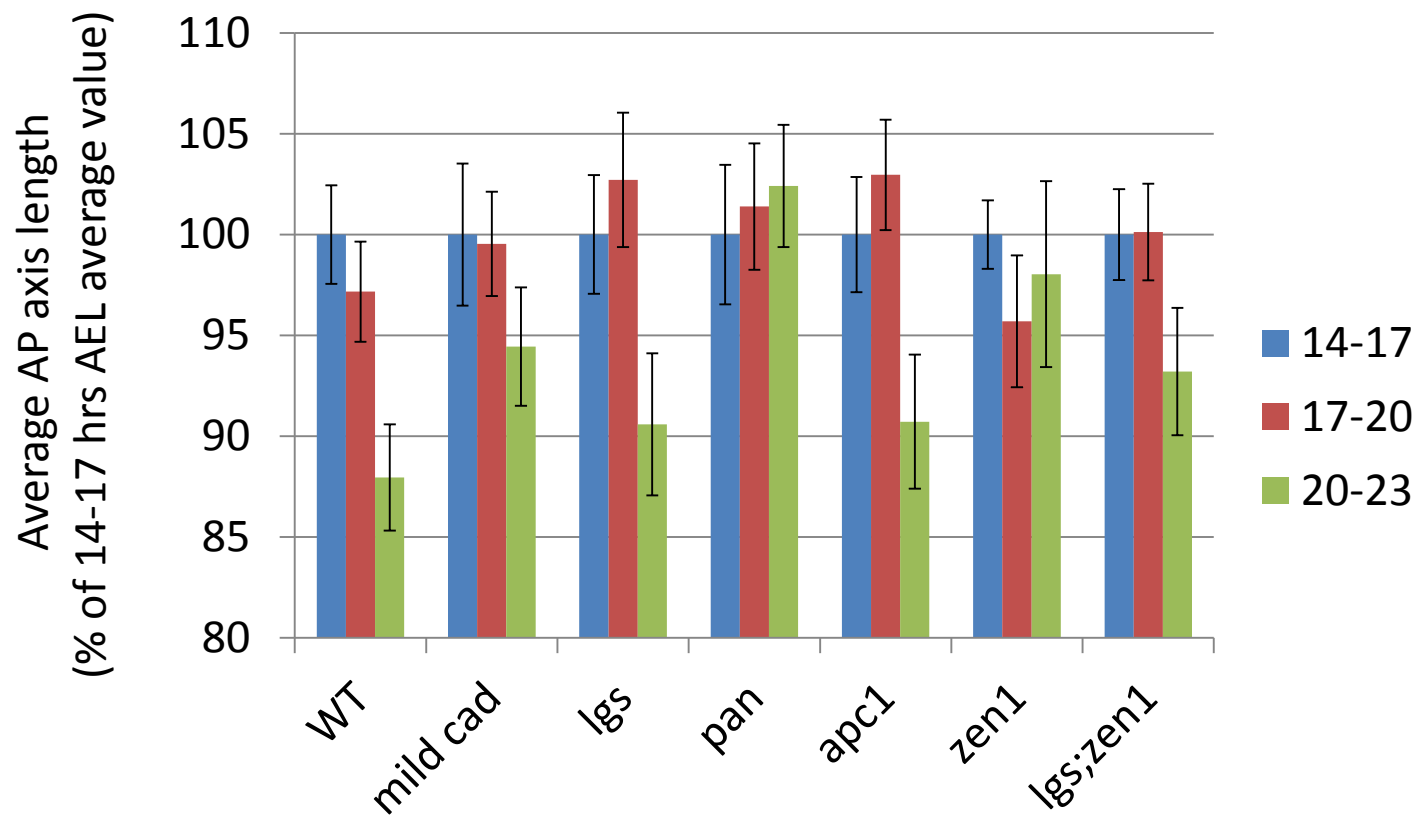

Supplement: Figure S5 — Average AP axis lengths over time for WT and RNAi knockdowns. For 14–17, 17–20, 20–23 hours AEL egg collections, the average AP axis lengths were calculated and normalized to 14–17 hours AEL average value for WT, mild Tc-cad, Tc-lgs, Tc-pan, Tc-apc1, Tc-zen1, and Tc-lgs;Tc-zen1 RNAi. (PDF) [file pgen.1004677.s005.pdf]
