# Supplementary material for: Caudal Regulates the Spatiotemporal Dynamics of Pair-Rule Waves in Tribolium
Source: PLoS Genet. 2014 Oct 16;10(10):e1004677. doi: 10.1371/journal.pgen.1004677 (PMC4199486; doi:10.1371/journal.pgen.1004677)
Supplement: Figure S3 — Tc-eve in severe and mild Tc-cad knockdowns. (A) Shown are two embryos with comparable stage (flattened posterior stage); Tc-eve is expressed in two stripes in WT while its expression is abolished in strong Tc-cad RNAi. (B) In mild Tc-cad RNAi, Tc-eve stripes split into two secondary stripes (upper embryo; similar to WT; see class III.1 embryo in Figure 3 A) in some embryos, while in other embryos they do not (lower embryo). Anterior to left. (PDF) [file pgen.1004677.s003.pdf]

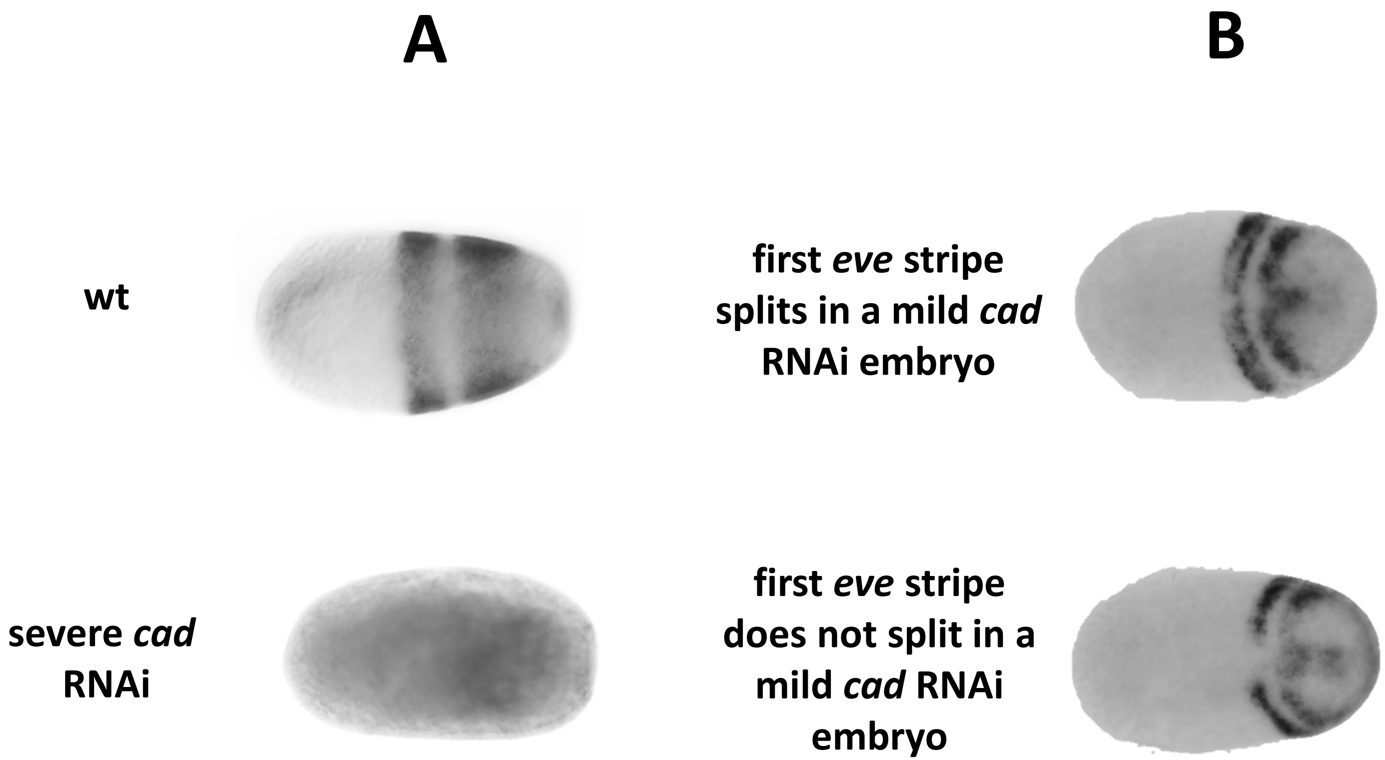
A
B
wt
first *eve* stripe splits in a mild *cad* RNAi embryo
severe *cad* RNAi
first *eve* stripe does not split in a mild *cad* RNAi embryo
