# Supplementary material for: Caudal Regulates the Spatiotemporal Dynamics of Pair-Rule Waves in Tribolium
Source: PLoS Genet. 2014 Oct 16;10(10):e1004677. doi: 10.1371/journal.pgen.1004677 (PMC4199486; doi:10.1371/journal.pgen.1004677)
Supplement: Text S1 — Matlab code for Movies S1, S2, S3, S4, S5, S6. (DOCX) [file pgen.1004677.s016.docx]

Text S1 Matlab code for Movies S1-S6.

clear

clc

close all

%temporal resolution: affects only visualization, not results

dt= 0.004;

%spatial resolution: affects only visualization, not results

dx= 1e-4;

%time axis: '0' is at Tc-eve expression initiation, and '1' is

%the end of the blastoderm stage.

t= 0:dt:1;

%x-axis: '0' is anterior end and '1' is posterior end

x= 0:dx:1;

%the maximum frquency (=number of stripes)

%observed in the blastoderm: 2.7 stripes per simulation time

f=2.7;

%a placeholder for the static frequency gradient of both backgrounds

static_freq_gradient=cell(2,1);

%a placeholder for the frequency gradient of both backgrounds through time

%(useful in case of simulating gradient dynamics)

dynamic_freq_gradient=cell(2,length(t));

for n=1:2

%Enter first and second backgrounds you want to compare

%For wt, enter 'wt' (enter 'wt' with quotation, same below)

%For wt without buildup phase, enter 'wt no buildup'

%For lgs, enter 'lgs'

%For pan, enter 'pan'

%For apc, enter 'apc'

%For zen, enter 'zen'

%For lgs;zen, enter 'lgs;zen'

bkg{n}=input('Enter first backgroud: ');

if(strcmp(bkg{n},'wt'))

%params of wt frequency gradient

slope(n)=2.6;

intercept(n)=.4;

buildup_rate(n)=5;

elseif(strcmp(bkg{n},'wt no buildup'))

%params of wt frequency gradient without buildup phase

slope(n)=2.6;

intercept(n)=.4;

buildup_rate(n)=inf;

elseif(strcmp(bkg{n},'lgs'))

%params of lgs frequency gradient

slope(n)=2;

intercept(n)=.6;

buildup_rate(n)=5;

elseif(strcmp(bkg{n},'pan'))

%params of pan frequency gradient

slope(n)=1;

intercept(n)=.25;

buildup_rate(n)=5;

elseif(strcmp(bkg{n},'apc'))

%params of apc frequency gradient

slope(n)=2.6;

intercept(n)=.25;

buildup_rate(n)=4;

elseif(strcmp(bkg{n},'zen'))

%params of zen frequency gradient

slope(n)=2.6;

intercept(n)=.25;

buildup_rate(n)=5;

else

elseif(strcmp(bkg{n},'lgs;zen'))

%params of lgs;zen frequency gradient

slope(n)=2;

intercept(n)=.52;

buildup_rate(n)=5;

else

error('invalid input!')

end

static_freq_gradient{n}=f*(slope(n)*(x-intercept(n)));

static_freq_gradient{n}(static_freq_gradient{n}<0)=0;

static_freq_gradient{n}(static_freq_gradient{n}>f)=f;

%creating a placeholder for eve oscillation phases in space and time

phase{n}= zeros(length(t),length(x));

%initializing eve phases: .95*pi means the oscillators are about to turn

%ON (=phase>pi, where oscillator output=-sin(phase))

phase{n}(1,:)= 0.95*pi;

end

for n=1:2

dynamic_freq_gradient{n,1}=...

(1-exp(-buildup_rate(n)*t(1)))*static_freq_gradient{n};

for nt=2:length(t)

dynamic_freq_gradient{n,nt}=...

(1-exp(-buildup_rate(n)*t(nt)))*static_freq_gradient{n};

phase{n}(nt,:)=...

phase{n}(nt-1,:)+...

dynamic_freq_gradient{n,nt}*dt*2*pi;

end%for nt

%translating phase into expression values

wave{n}= -sin(phase{n});

%turning sine signal into square

wave{n}(wave{n}<=0)=0;

wave{n}(wave{n}>0)=1;

end%for n

%plotting the spatiotemporal expression of Tc-eve (blue) and the

%frequency gradient (red)

for nt=1:length(t)

for n=1:2

subplot(strcat('21',num2str(n)))

area(x,wave{n}(nt,:));%plotting Tc-eve expression

hold on

plot(x,dynamic_freq_gradient{n,nt}/f,'r','LineWidth',5)%plotting the frequency gradient

axis([0 1 0 1])

set(gca,'XTick',[])

title(bkg{n})

pause(1e-50)

hold off

end

end
