# Supplementary material for: Caudal Regulates the Spatiotemporal Dynamics of Pair-Rule Waves in Tribolium
Source: PLoS Genet. 2014 Oct 16;10(10):e1004677. doi: 10.1371/journal.pgen.1004677 (PMC4199486; doi:10.1371/journal.pgen.1004677)
Supplement: Text S2 — Matlab code for Movies S7, S8, S9, S10. (DOCX) [file pgen.1004677.s017.docx]

Text S2 Matlab code for Movie S7-S10.

%Simulation of clock and wavefront model with noisy frequency gradient

%levels in 2D

clear

clc

close all

%temporal resolution

dt= 0.001;

%spatial resolution

dx= 2e-3;

dy= 2e-3;

%time axis

t= 0:dt:.3;

%x axis

x= 0:dx:1;

%y axis

y= 0:dy:.2;

%maximum frequency

f= 50;

%placeholder for oscillator phases across space and time

phase= zeros(length(x),length(y));

%placeholder for the frequency gradient space and time

freq_gradinet= zeros(length(x),length(y));

%velocity

v=3;

%level noise

q=input('Level noise? (enter a number >0 for yes): ');

if(q>0)

level_noise_strength=2.5;

else

level_noise_strength=0;

end

%slope of frequency gradient

slope= 10;

%thresholded or not?

thresholded= input('Thresholded? (enter a number >0 for yes): ');

if(thresholded<0)

thresholded=0;

end

f_thresh= f/2;

shift=0;

for nt=2:length(t)

%posterior shift of gradient per dt

shift=shift+.005;

for ny=1:length(y)

level_noise=level_noise_strength*rand(1,length(x));

freq_gradient(:,ny)=...

level_noise +...

f*(1./(1+exp(-slope*(x-shift))));

effective_freq_gradient=freq_gradient;

if(thresholded)

effective_freq_gradient(effective_freq_gradient>=f_thresh)=f;

effective_freq_gradient(effective_freq_gradient<f_thresh)=0;

end

for nx=1:length(x)

phase(nx,ny)= phase(nx,ny)+ effective_freq_gradient(nx,ny)*dt*2*pi;

end%for nx

end%for ny

wave= sin(-phase);

wave(wave<=0)=0;

wave(wave>0)=1;

%plotting

if(thresholded)

I=[freq_gradient'/f; effective_freq_gradient'/f; wave'];

else

I=[freq_gradient'/f; wave'];

end

I=imresize(I,size(I)*2);

imshow(I);

pause(1e-20)

end%for nt
