# Supplementary material for: Caudal Regulates the Spatiotemporal Dynamics of Pair-Rule Waves in Tribolium
Source: PLoS Genet. 2014 Oct 16;10(10):e1004677. doi: 10.1371/journal.pgen.1004677 (PMC4199486; doi:10.1371/journal.pgen.1004677)
Supplement: Text S3 — Supplemental experimental procedures. (DOCX) [file pgen.1004677.s018.docx]

Text S3 Supplemental Experimental Procedures

*Comparing Tc-cad gradients in two egg collections*

To compare the Tc-cad gradient in two different egg collections (either from two different time windows, from WT versus knockdown, or from two different knockdowns), we simultaneously fixed and performed chromogenic in situ staining for the two egg collections to show Tc-cad expression. We specified a box (yellow in Figure 1 E and Figure 2 A, C, E, G, I, K) of enough width to span most of the AP axis (away from out of focus edges) and of enough height for the box to be confined within the embryo across the whole AP axis. We then calculated the average intensity of each one-pixel-width column of the box, to get a one dimensional array holding Tc-cad intensity values across the AP axis (using ImageJ). We then fitted (least squares, using Matlab) Tc-cad intensity values to linear-with-plateau curves (defined by the three curve descriptors in Figure 1 F), and calculated the average values of the 3 curve descriptors for each egg collection. We finally normalized the average value of one egg collection to the other.

*Calculating class distribution graphs and class durations*

Class distribution graphs (Figure 5 A, B, C, D, E, F) were created by counting the embryos belonging to Tc-eve oscillation cycles I, II, III, and >III in each of the 3-hour developmental windows: 14-17, 17-20, 20-23, and 23-26 hours AEL. The aberrant germband formation in Tc-apc1, Tc-zen1, and Tc-lgs;Tc-zen1 RNAi embryos hindered the examination of *Tc-eve* expression in 23-26 hours AEL egg collections. Class durations (Figure 5 A’, B’, C’, D’, E’, F’) were calculated from the corresponding class distribution graphs (Figure 5 A, B, C, D, E, F, respectively) by averaging the percentage of the occurrence of each class across all examined timed egg collections. This is equivalent to combining all egg collections into one large collection, spanning 14-26 hours AEL (in Figure 5 A, B, C) or 14-23 hours AEL (in Figure 5 D, E, F), then using the overall percentage of embryos in each class as an estimate of its duration. Percentages rather than absolute numbers are used in calculations to correct for differences in the number of eggs in each collection.

*Performing spatial measurements of Tc-eve expression*

For each embryo within a timed egg collection in situ stained to show Tc-eve expression, three measurements were made and normalized to embryo AP length: position of Tc-eve anterior border, width of first Tc-eve stripe, and width of second Tc-eve stripe. At late blastoderm stage, the embryo undergoes morphogenetic movements that can affect the faithfulness of measurements. However, we previously showed that no significant cell movements along AP axis take place during the blastoderm stage except for a posterior flattening of the embryo (El-Sherif et al., Development, 2012). To characterize this flattening, we measured the AP axis length for WT as well as RNAi knockdowns considered in this study over time (Figure S5). We found that posterior flattening mostly takes place during 20-23 hours AEL in WT and mild *Tc-cad*, *Tc-lgs*, *Tc-apc1*, and *Tc-lgs*;*Tc-apc1* RNAi embryos, and leads to a reduction of AP axis length by 10%. To compensate for this reduction, we, therefore, multiplied all *Tc-eve* expression measurements at 20-23 hours AEL by 0.9 only for WT and mild *Tc-cad*, *Tc-lgs* ,*Tc-apc1*, and *Tc-lgs*;*Tc-apc1* RNAi embryos. For *Tc-pan* and *Tc-zen1* RNAi embryos, we did not detect a significant reduction in AP axis length (Figure S5), so we did not employ any corrections for them.
